# Surgical Management of Sigmoid Volvulus: A Retrospective Review of Six Cases with a Focus on the Sharon Operation

**DOI:** 10.70352/scrj.cr.25-0487

**Published:** 2026-01-10

**Authors:** Keisuke Inoue, Tetsu Yamamoto, Takahito Taniura, Kazunari Ishitobi, Ayana Kishimoto, Shunsuke Kaji, Takayuki Tanaka, Takeshi Matsubara, Masaaki Hidaka

**Affiliations:** Department of Digestive and General Surgery, Shimane University Faculty of Medicine, Izumo, Shimane, Japan

**Keywords:** Sharon operation, sigmoid volvulus, older patients, elective colectomy, minimally invasive surgery

## Abstract

**INTRODUCTION:**

Sigmoid volvulus is a clinically significant cause of large bowel obstruction that occurs particularly in older patients with a reduced physical function. Although endoscopic detorsion is the first-line treatment, volvulus recurrence is common and often requires elective surgery. Despite various reports on the surgical procedures, the optimal approach remains controversial. The Sharon operation, first introduced in 1985, is a minimally invasive technique that avoids mobilization of the sigmoid colon via a small incision in the left lower quadrant. Although it offers several advantages, such as shorter operative time, minimal invasiveness, and favorable clinical outcomes, it remains underutilized, especially in Japan. We evaluated the clinical utility and indications of the Sharon operation in high-risk patients with recurrent sigmoid volvulus.

**CASE PRESENTATION:**

This retrospective study analyzed 6 patients who underwent surgery for sigmoid volvulus at our institution between 2015 and 2023. The median age was 82.5 years, and all patients had a prior history of volvulus. Four patients underwent elective surgery following successful endoscopic detorsion, and 2 underwent emergency surgery due to suspected bowel necrosis. Among the elective cases, 2 received laparoscopic sigmoidectomy and 2 underwent the Sharon operation. The Sharon operation group had a shorter operative time (mean 74 min) than the laparoscopic surgery group (mean 225.5 min), with minimal blood loss in both groups. The only postoperative complication was superficial surgical site infection (SSI) in 1 laparoscopic case. During a median follow-up period of 61 months, no recurrence was observed. We chose the Sharon operation preferentially for patients with advanced age, poor nutritional status, or severe comorbidities including cardiac dysfunction.

**CONCLUSIONS:**

The Sharon operation is a safe and effective option for recurrent sigmoid volvulus, particularly in older patients or those with significant comorbidities. Considering its shorter operative time and minimal incision, it may be helpful in patients who are unsuitable for standard laparoscopic or open procedures. No recurrence was observed during long-term follow-up, suggesting a potentially curative outcome of the Sharon operation.

## Abbreviations

PNI
: prognostic nutritional index
SSI
: surgical site infection

## INTRODUCTION

Colonic volvulus is a relatively uncommon condition; however, it is the third most common cause of colonic obstruction.^1)^ It is the most common type of colonic volvulus and typically occurs in older patients with low physical activity or psychiatric disorders.^2)^ The elongation of the sigmoid colon is thought to cause sigmoid volvulus and the elongated sigmoid colon is prone to torsion.

Emergency surgery is required when bowel necrosis is suspected, and sigmoid colon resection with colostomy (Hartmann’s operation) is usually considered according to the patient's background, general condition, and intraoperative bowel findings.^3)^ Endoscopic decompression and detorsion are the first choices for sigmoid volvulus with no evidence of bowel ischemia. However, recurrence is common in patients treated endoscopically. Therefore, elective surgery should be considered, particularly in patients with recurrent sigmoid volvulus.^4)^

Although various surgical techniques have been reported for sigmoid volvulus, there is no consensus on the optimal surgical approach, particularly in terms of outcome and operative time. Procedures such as mesosigmoidoplasty or colopexy have a lower risk of postoperative complications; however, they are associated with a higher risk of recurrence. Therefore, resection of the redundant sigmoid colon should be performed, whenever possible.^5,6)^ However, sigmoid volvulus often occurs in older patients with severe and multiple comorbidities. Therefore, many patients are not suitable candidates for standard laparoscopic or open surgery.

The Sharon operation, first described in 1985, is a technique that avoids mobilization of the sigmoid colon via a small incision in the left lower quadrant. Although it offers advantages such as shorter operative time and minimal invasiveness, it remains uncommon in Japan.^7)^

Although the Sharon procedure was first reported nearly 40 years ago, its adoption in Japan has been limited. We speculated on the reasons for this. First, laparoscopic techniques for colorectal diseases, especially for colorectal cancer, have rapidly become widespread and standardized as minimally invasive surgery. Second, laparoscopic surgery has significant advantage that surgeons can observe the abdominal cavities including the presence of adhesions and the relationship with adjacent organs.

We herein report our surgical experience with sigmoid volvulus, focusing on the Sharon operation, and evaluating its clinical features and surgical outcomes.

## CASE PRESENTATION

### Patients and methods

We retrospectively analyzed 6 patients who underwent surgical treatment for sigmoid volvulus in our hospital between 2015 and 2023. Patient characteristics, surgical outcomes, and short-term outcomes were analyzed. Postoperative follow-up was conducted at our hospital for at least 6 months as an outpatient; thereafter, follow-up was performed at each patient’s local hospital. To confirm the general condition or recurrence, telephone contact was performed with the patients or family in 5 of 6 cases.

### Indication for surgical procedures

During the study period, the standard elective surgical procedure for sigmoid colon volvulus at our institution was laparoscopic sigmoidectomy. However, for severe condition patients, such as severe cardiac disease or extremely older, the Sharon operation was considered because of the advantage of less invasiveness.

### Procedure for the Sharon operation

**Fig. 1:**
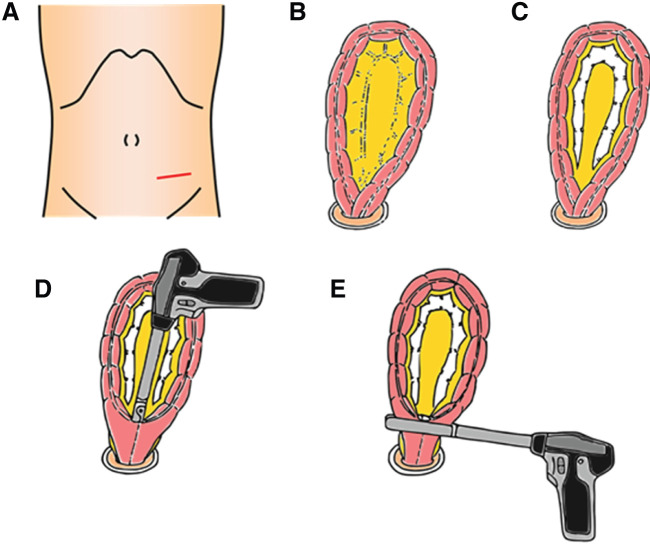
Surgical procedure of the sharon operation. (**A**) A small left-sided McBurney incision is made to access the abdominal cavity. (**B**) The redundant sigmoid colon is elevated through a mini-laparotomy. (**C**) The redundant sigmoid colon mesentery is divided along with the tract. (**D**) Side-to-side anastomosis is performed using a linear stapler. (**E**) The redundant sigmoid colon is resected and the entry hole is closed using a linear stapler.

**Fig. 2:**
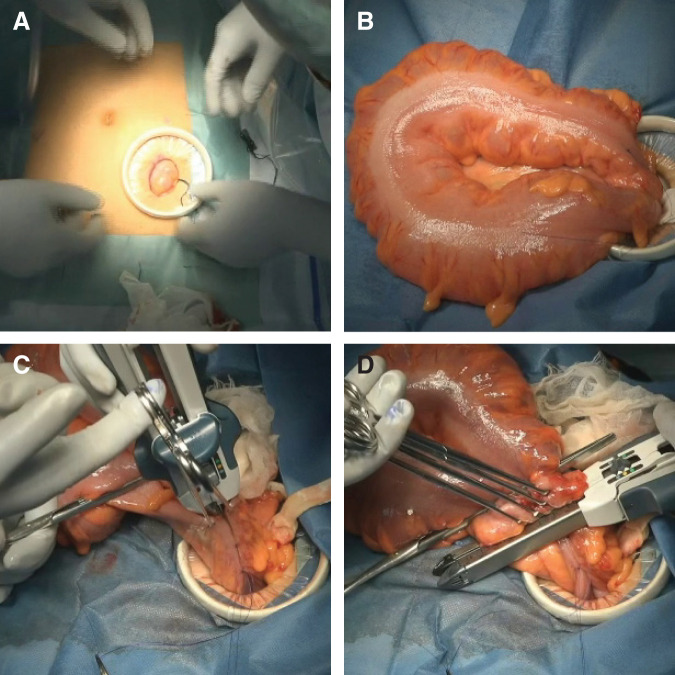
Intraoperative steps of the Sharon operation. (**A**) Performing a small left lower quadrant mini-laparotomy. (**B**) Exteriorizing the redundant sigmoid colon through the mini-laparotomy. (**C**) Performing side-to-side anastomosis using a linear stapler. (**D**) Resecting the redundant sigmoid colon with stapled enterotomy closure.

Under general or epidural anesthesia, the patient was placed in the supine position. A 4-cm left lower quadrant pararectal incision was made to access the abdominal cavity. In most cases, the redundant sigmoid colon is recognized directly beneath the incision and can easily be exteriorized. The sigmoid colon was drawn as far as possible, and the resection line was determined near the wound margin. The mesentery of the sigmoid colon was divided based on the planned resection level. Functional end-to-end anastomosis was performed using a linear stapler and the anastomosed bowel was returned to the abdominal cavity. The abdominal wall was then closed (**Fig. 1**). Representative intraoperative photographs illustrating these steps are shown in **Fig. 2**.

### Results

Patient characteristics are shown in **Table 1**. The median age was 82.5 years (range 54–92 years), with a male-to-female ratio of 5:1. All 6 patients (100%) had a history of sigmoid volvulus. Initial symptoms included abdominal pain in 4 patients and abdominal distension in 2 patients; none presented with hematochezia. Four patients (66.7%) had a history of multiple episodes of volvulus and underwent elective surgery, while 2 patients (33.3%) required emergency surgery due to suspected intestinal necrosis. The median interval from initial consultation to surgery was 5 months (range 2–120 months).

**Table 1:** Cases involving surgical management of sigmoid volvulus at our hospital

Case	Sex	Age	Operative procedure	Endoscopic examination	Interval from onset to surgery	Comorbidities	PNI
1	M	76	Sigmoidectomy	Intestinal necrosis	Emergency operation	Syringomyelia	39.05
2	M	92	Sharon operation	Endoscopic reduction	7 days	Severe aortic valve stenosis	38.25
3	M	85	Sigmoidectomy	Intestinal necrosis	Emergency operation	Dementia	44.55
4	M	82	Laparoscopic sigmoidectomy	Endoscopic reduction	17 days	Dementia	42.95
5	M	83	Laparoscopic sigmoidectomy	Endoscopic reduction	78 days	None	42.15
6	F	54	Sharon operation	Endoscopic reduction	178 days	Severe bradycardia Mitral regurgitation	48.30

PNI, prognostic nutritional index

Endoscopic detorsion was performed in 4 patients as the first treatment, with a median time to surgery following successful detorsion of 47.5 days (range 7–178 days). The median Charlson Comorbidity Index was 6.5 (range 2–9); 2 patients had severe cardiac disease, 1 patient had a neurological disorder, and 1 patient had significant malnutrition. The median PNI was 42.6 (range 38.3–48.3), with 2 patients (33.3%) having a low PNI (<40).

The details of elective surgeries are shown in **Table 2**. Two cases each of laparoscopic sigmoid colon resection and the Sharon operation were performed. The mean operation time was 225.5 min, (range 203–248 min) for laparoscopic surgery and 74 min (range 59–89 min) for the Sharon operation. Blood loss was minimal in both the groups. None of the patients required a stoma creation. Postoperative complications occurred in 1 case of laparoscopic sigmoidectomy, which was a superficial SSI that was treated with incisional drainage (Clavien–Dindo grade I). The mean postoperative hospital stay was 14.5 days (range 14–15 days) for laparoscopic surgery and 13 days (range 10–16 days) for the Sharon operation. The median follow-up duration was 61 months (range 22–86 months) with no recurrence. Two surgeons performed the Sharon procedure; both had over 10 years of experience in colorectal surgery and were Board Certified Surgeons in Gastroenterology by the Japanese Society of Gastroenterological Surgery.

**Table 2:** Outcomes of elective surgery for sigmoid volvulus at our hospital

Case	Sex	Age	Comorbidities	Laparoscopic/Open	Procedure	Time (min)	Blood loss	Complications	Postoperative hospital stay (days)
2	M	92	Severe aortic valve stenosis	Open	Sharon operation	89	Minimal	None	16
4	M	82	Dementia	Laparoscopic	Sigmoidectomy	248	Minimal	Superficial SSI	15
5	M	83	None	Laparoscopic	Sigmoidectomy	203	Minimal	None	14
6	F	54	Severe bradycardia Mitral regurgitation	Open	Sharon operation	59	Minimal	None	10

SSI, surgical site infection

## DISCUSSION

Colonic volvulus, first reported in 1836, is a condition in which a segment of the colon twists around the mesentery, potentially causing obstruction.^8)^ It accounts for approximately 5%–42% of all cases of intestinal obstruction. In previous reports, the sigmoid colon was the most frequent site (60%–75%), followed by the cecum (25%–40%), transverse colon (1%–4%), and splenic flexure (1%).^2,9)^ Sigmoid volvulus is the third most common cause of colonic obstruction, following colorectal cancer and diverticulitis.^1)^ Older people, particularly those > 60 years of age, are at a higher risk of developing sigmoid volvulus. Sigmoid volvulus has a high recurrence rate, and repeated episodes carry the risk of complications and mortality.^10,11)^ Risk factors include chronic constipation, high-fiber diet, laxative use, diabetes mellitus, psychiatric disorders, and prolonged bed rest.^9)^ Anatomical predisposition, such as a redundant sigmoid colon, is also recognized, and no significant sex difference has been reported.^2)^

Pathogenesis typically involves the torsion of a redundant sigmoid colon. When rotation exceeds 180°, it may lead to intestinal obstruction, ischemia, or necrosis, potentially resulting in perforation. In contrast, spontaneous resolution has been reported in approximately 2% of cases.^12)^ Approximately 70% of cases show counterclockwise rotation, but the reason for counterclockwise rotation remains unclear.^13)^

Management strategies for sigmoid volvulus are divided into nonoperative and surgical approaches. Endoscopic detorsion is generally recommended when a CT scan or other imaging modality shows ischemia or perforation. Previous studies have reported success rates ranging from 60% to 95%, with a complication rate of 4% and a mortality rate of 3%.^4,14)^

Although the effectiveness of decompression tube placement and bowel irrigation has been reported, the standard duration of placement remains unclear. In addition, the recurrence rate after endoscopic detorsion remains high (67%–75%).^15–18)^

Surgical treatments include emergency and elective surgery. Approximately 5%–25% of patients present with signs of ischemia, perforation, peritonitis, or septic shock at the initial evaluation, and require emergency surgery.^9)^ The decision to perform bowel resection depends on the degree of ischemia. Because of the high risk of delayed necrosis or perforation, bowel resection should be considered even if the ischemic change is minimal.

Hartmann’s procedure was commonly performed in these cases. A postoperative complication rate of 8% and mortality rate of approximately 5% have been reported. The anastomotic leakage rate with primary anastomosis is reported to be 7%, which is relatively high in comparison to standard colectomy.^19)^ Elective surgery is considered when endoscopic detorsion and decompression are successful. Various procedures have been described including sigmoid colectomy, mesosigmoidoplasty, and sigmoidopexy. However, some studies have reported a recurrence rate of 4% after mesosigmoidoplasty. Another study mentioned that there is no evidence supporting the prevention of future torsion by sigmoidopexy.^5,6)^ Therefore, resection of the redundant colon should be considered whenever feasible. Sigmoid volvulus occurs frequently in older patients, many of whom have significant comorbidities. In cases with an impaired cardiac or pulmonary function, endoscopic treatment may be preferred to avoid surgery. However, recurrent volvulus should be promptly considered for surgical intervention. Older patients tend to delay initial consultation owing to a lack of subjective symptoms, which sometimes results in life-threatening complications such as peritonitis or septic shock.^20)^ Therefore, early surgical consideration is essential to prevent such outcomes.

Because many high-risk patients exist in this population, a short and minimally invasive surgery is desirable. The Sharon operation can fulfill these requirements.

The Sharon operation, first described in 1985, involves a transverse incision via a left-sided McBurney-style skin incision, which allows easy access for sigmoid colon resection. It is a simple technique that can be performed through a small, approximately 4-cm incision.^7)^ Although reported in a limited number of cases, the operative time for the Sharon operation is typically within 90 min, with minimal postoperative complications and no recurrence.

In this study, the outcomes of the Sharon operation were comparable to previous reports regarding operative times and blood loss.^21)^ No perioperative complications were observed, and the postoperative course was uneventful. Additionally, we could perform a Sharon operation for the patients who are extremely older or have severe cardiac dysfunction, who have to avoid laparoscopic surgery, indicating that this procedure is less invasive than laparoscopic surgery. Although the Sharon operation can be performed through a small incision, this makes observation of the abdominal cavity or management of intra-abdominal adhesions difficult. These are considered disadvantages of the Sharon procedure. However, all sigmoid volvulus cases had no intra-abdominal severe adhesion despite long periods of illness. Sigmoid volvulus commonly arises in a redundant, freely mobile sigmoid colon; therefore, cases with dense adhesions to surrounding organs or the abdominal wall are thought to be less likely to develop volvulus. In particular, successful endoscopic detorsion suggests sigmoid colon mobility and may indicate suitability for the Sharon operation. Conversely, in emerging surgery due to the failure of endoscopic detorsion, marked torsion, or suspicion of ischemia, patients may not be candidates for the Sharon procedure. When intraperitoneal adhesions are anticipated, particularly in patients with previous abdominal surgery, laparoscopic observation can be used to inspect the abdominal cavity and, if necessary, to perform safe adhesiolysis.^22)^

In Japan, laparoscopic sigmoidectomy is widely used as the standard elective approach for sigmoid volvulus. However, older patients with severe comorbidities are not always optimal candidates for laparoscopic surgery. Given its short operative time and minimal incision, the Sharon operation may offer a practical option for high-risk patients in current Japanese practice. Our favorable outcomes support this role.

## CONCLUSIONS

Sigmoid volvulus frequently occurs in older patients and often recurs, even after successful endoscopic detorsion. Surgical intervention should be considered in recurrent cases. However, these patients are generally at high risk for surgery because of significant complications and a reduced organ function. The Sharon operation features a minimally invasive approach and short operative time and may be particularly suitable for older or cardiopulmonary-compromised patients with recurrent sigmoid volvulus. Based on our experience, the Sharon operation offers a safe and effective alternative to conventional colectomy and may help prevent recurrence with a minimal postoperative burden.

## DECLARATIONS

### Availability of data and materials

The datasets supporting the conclusions of this article are provided within the manuscript.

## References

[1] Ballantyne GH, Brandner MD, Beart RW Jr., et al. Volvulus of the colon: incidence and mortality. Ann Surg 1985; 202: 83–92. 4015215 10.1097/00000658-198507000-00014 PMC1250842

[2] Perrot L, Fohlen A, Alves A, et al. Management of the colonic volvulus in 2016. J Visc Surg 2016; 153: 183–92. 27132752 10.1016/j.jviscsurg.2016.03.006

[3] Bagarani M, Conde AS, Longo R, et al. Sigmoid volvulus in West Africa: a prospective study on surgical treatments. Dis Colon Rectum 1993; 36: 186–90. 8425424 10.1007/BF02051177

[4] Turan M, Sen M, Karadayi K, et al. Our sigmoid colon volvulus experience and benefits of colonoscope in detortion process. Rev Esp Enferm Dig 2004; 96: 32–5. 14971995 10.4321/s1130-01082004000100005

[5] Subrahmanyam M. Mesosigmoplasty as a definitive operation for sigmoid volvulus. Br J Surg 1992; 79: 683–4. 1643486 10.1002/bjs.1800790731

[6] Akgun Y. Mesosigmoplasty as a definitive operation in treatment of acute sigmoid volvulus. Dis Colon Rectum 1996; 39: 579–81. 8620812 10.1007/BF02058715

[7] Sharon N, Efrat Y, Charuzi I. A new operative approach to volvulus of the sigmoid colon. Surg Gynecol Obstet 1985; 161: 483–4. 4049218

[8] Tan PY, Corman ML. History of colonic volvulus. Semin Colon Rectal Surg 1999; 10: 122–8.

[9] Tian BWCA, Vigutto G, Tan E, et al. WSES consensus guidelines on sigmoid volvulus management. World J Emerg Surg 2023; 18: 34. 37189134 10.1186/s13017-023-00502-x PMC10186802

[10] Halabi WJ, Jafari MD, Kang CY, et al. Colonic volvulus in the United States: trends, outcomes, and predictors of mortality. Ann Surg 2014; 259: 293–301. 23511842 10.1097/SLA.0b013e31828c88ac

[11] Atamanalp SS. Sigmoid volvulus: diagnosis in 938 patients over 45.5 years. Tech Coloproctol 2013; 17: 419–24. 23224856 10.1007/s10151-012-0953-z

[12] Ballantyne GH. Review of sigmoid volvulus. Clinical patterns and pathogenesis. Dis Colon Rectum 1982; 25: 823–30. 6293790 10.1007/BF02553326

[13] Shepherd JJ. The epidemiology and clinical presentation of sigmoid volvulus. Br J Surg 1969; 56: 353–9. 5781046 10.1002/bjs.1800560510

[14] Ören D, Atamanalp SS, Aydinli B, et al. An algorithm for the management of sigmoid colon volvulus and the safety of primary resection: experience with 827 cases. Dis Colon Rectum 2007; 50: 489–97. 17205203 10.1007/s10350-006-0821-x

[15] Dülger M, Cantürk NZ, Utkan NZ, et al. Management of sigmoid colon volvulus. Hepatogastroenterology 2000; 47: 1280–3. 11100333

[16] Hattori S, Aramaki O, Watanabe Y, et al. Transanal Decompression tube placement for treatment of sigmoid volvulus. J Anus Rectum Colon 2024; 8: 305–15. 39473707 10.23922/jarc.2024-027 PMC11513424

[17] Ifversen AK, Kjaer DW. More patients should undergo surgery after sigmoid volvulus. World J Gastroenterol 2014; 20: 18384–89. 25561806 10.3748/wjg.v20.i48.18384 PMC4277976

[18] Bruzzi M, Lefèvre JH, Desaint B, et al. Management of acute sigmoid volvulus: short- and long-term results. Colorectal Dis 2015; 17: 922–8. 25808350 10.1111/codi.12959

[19] Kuzu MA, Aşlar AK, Soran A, et al. Emergent resection for acute sigmoid volvulus: results of 106 consecutive cases. Dis Colon Rectum 2002; 45: 1085–90. 12195194 10.1007/s10350-004-6364-0

[20] Atamanalp SS, Ozturk G. Sigmoid volvulus in the elderly: Outcomes of a 43-year, 453-patient experience. Surg Today 2011; 41: 514–9. 21431484 10.1007/s00595-010-4317-x

[21] Honjo H, Mike M, Kusanagi H. Sharon operation in treatment of sigmoid volvulus (in Japanese with English abstract). Nihon Shokaki Geka Gakkai Zasshi (Jpn J Gastroenterol Surg) 2017; 50: 334–8.

[22] Minagawa Y, Ishiyama Y, Fukuda T, et al. A case of sigmoid colon volvulus with transanal ileus tube placement and Sharon’s operation performed safely. J Surg Case Rep 2022; 2022: rjac429. 36381983 10.1093/jscr/rjac429 PMC9643033

